# Comparison of equations for estimating glomerular filtration rate in screening for chronic kidney disease in asymptomatic black Africans: a cross sectional study

**DOI:** 10.1186/s12882-017-0788-y

**Published:** 2017-12-20

**Authors:** Geoffrey Omuse, Daniel Maina, Jane Mwangi, Caroline Wambua, Alice Kanyua, Elizabeth Kagotho, Angela Amayo, Peter Ojwang, Rajiv Erasmus

**Affiliations:** 1 Department of Pathology, Aga Khan University Hospital Nairobi, P.O. Box 30270-00100, Nairobi, Kenya; 2 PathCare Kenya Ltd., P.O. Box 12560-00606, Nairobi, Kenya; 3 Department of Pathology, Aga Khan Hospital, P.O. Box 2289, Dar es Salaam, Tanzania; 4 Department of Human Pathology, University of Nairobi, P.O. Box 19676-00200, Nairobi, Kenya; 5 Department of Pathology, Maseno University, P.O. Box Private Bag, Maseno, Kenya; 6 Division of Chemical Pathology, Department of Pathology, Stellenbosch University, P.O. Box 19113, Tygerberg Hospital, Cape Town, South Africa

**Keywords:** MDRD, CKD-EPI, Cockcroft-Gault, FAS, eGFR

## Abstract

**Background:**

Several equations have been developed to estimate glomerular filtration rate (eGFR). The common equations used were derived from populations predominantly comprised of Caucasians with chronic kidney disease (CKD). Some of the equations provide a correction factor for African-Americans due to their relatively increased muscle mass and this has been extrapolated to black Africans. Studies carried out in Africa in patients with CKD suggest that using this correction factor for the black African race may not be appropriate. However, these studies were not carried out in healthy individuals and as such the extrapolation of the findings to an asymptomatic black African population is questionable. We sought to compare the proportion of asymptomatic black Africans reported as having reduced eGFR using various eGFR equations. We further compared the association between known risk factors for CKD with eGFR determined using the different equations.

**Methods:**

We used participant and laboratory data collected as part of a global reference interval study conducted by the Committee of Reference Intervals and Decision Limits (C-RIDL) under the International Federation of Clinical Chemistry (IFCC). Serum creatinine values were used to calculate eGFR using the Cockcroft-Gault (CG), re-expressed 4 variable modified diet in renal disease (4v–MDRD), full age spectrum (FAS) and chronic kidney disease epidemiology collaboration equations (CKD-EPI). CKD classification based on eGFR was determined for every participant.

**Results:**

A total of 533 participants were included comprising 273 (51.2%) females. The 4v–MDRD equation without correction for race classified the least number of participants (61.7%) as having an eGFR equivalent to CKD stage G1 compared to 93.6% for CKD-EPI with correction for race. Only age had a statistically significant linear association with eGFR across all equations after performing multiple regression analysis. The multiple correlation coefficients for CKD risk factors were higher for CKD-EPI determined eGFRs.

**Conclusions:**

This study found that eGFR determined using CKD-EPI equations better correlated with a prediction model that included risk factors for CKD and classified fewer asymptomatic black Africans as having a reduced eGFR compared to 4v–MDRD, FAS and CG corrected for body surface area.

## Background

Routine reporting of estimated glomerular filtration rate (eGFR) by medical laboratories for every serum creatinine request has been encouraged as a way of screening for chronic kidney disease (CKD) and has been shown to increase the absolute number of appropriate referrals to nephrologists [1]. Patients with early CKD may be asymptomatic and therefore reporting eGFR may help in early detection and appropriate interventions to stop or reverse progression of the disease [2].

The use of eGFR equations instead of creatinine clearance (CrCl) is preferred due to the difficulties associated with accurately collecting a 24 h urine sample. The most popular equations for estimating GFR are Cockcroft-Gault (CG), 4 variable modified diet in renal disease (4v–MDRD) and chronic kidney disease epidemiology collaboration (CKD-EPI). The CG equation was derived from an inpatient population comprising predominantly male patients with CKD [3]. The equation doesn’t correct for race and one requires the patient’s height and weight to correct for body surface area (BSA) in order to accurately classify CKD. The 4v–MDRD equation was derived from a population with kidney disease comprising a predominantly white population. Despite the low percentage of African-Americans in this study, they determined a correction for race [4]. It has been assumed, given the ancestral linkage between African Americans and black Africans that this correction should apply to black Africans. However, a study carried out in South Africa that measured GFR in black South Africans with established CKD or risk factors for developing CKD concluded that eGFR based on the 4v–MDRD equation without correction for race better correlated with measured GFR (mGFR) and had less bias [5]. A similar study carried out in Ghana in a rural African population arrived at a similar conclusion, however, this study compared eGFR to creatinine clearance [6]. Both CG and 4v–MDRD have been shown to underestimate normal and high mGFR values with a greater negative bias seen with CG [7, 8]. One of the drawbacks of the 4v–MDRD equation is that it doesn’t accurately classify patients with early CKD, in particular those with values >60 ml/min/1.73m^2^. This is not surprising given that it was derived from patients with CKD who had a mean eGFR of 39.8 ml/min/1.73m^2^. The utility of this equation in screening for CKD in an asymptomatic patient with no known risk factors for CKD is questionable given that subjectively healthy individuals generally have an eGFR in the range where the 4v–MDRD equation is inaccurate. The CKD-EPI equation has been found to better correlate with mGFR especially in those with eGFR > 60 ml/min/1.73m^2^ [9]. In a study carried out in Kenya amongst HIV patients with a median mGFR of 115 ml/min/1.73m^2^, both CKD-EPI and 4v–MDRD performed better without correction for race with CKD-EPI estimates being more precise [10]. A new equation called the full age spectrum (FAS) equation has been developed by Pottel et al. which factors in correction for race, age and gender by including population specific mean or median serum creatinine values in the determination of eGFR. This equation has the advantage of being applicable across different age groups from children as young as 2 years to the elderly above 65 years of age. The equation is yet to be validated in a black African population and was derived from population datasets largely comprising Caucasians [11]. Most of the studies done in Africa that have concluded that correcting for race is not necessary for a black African population were carried out in populations comprising patients with CKD or at a high risk of developing CKD. The extrapolation of this finding to an asymptomatic population should not be done without evidence that this observation is consistent.

None of the eGFR equations that are routinely used were derived from a black African population and as such their appropriateness in assessing kidney function in this population needs to be evaluated. Since creatinine generation is determined primarily by muscle mass and dietary intake [12], it is reasonable to assume that the performance of eGFR equations will be influenced by the extent to which the body habitus of an African population differs from the reference populations used when deriving them. Given the paucity of data on the performance of these equations in a subjectively healthy population, we compared eGFR determined using various eGFR equations and assessed which eGFR values best correlated with known risk factors for CKD and the proportion of asymptomatic black Africans with no known risk factors for CKD classified as having a reduced or normal eGFR by each equation.

## Methods

We determined eGFR using single measurements of serum creatinine from Kenyans participating in a cross-sectional multicenter, multinational, global reference interval study which is part of an initiative by the Committee of Reference Intervals and Decision Limits (C-RIDL) under the auspices of the International Federation of Clinical Chemistry (IFCC). The aim of the study is to determine reference intervals for common laboratory tests across different geographical regions and populations, and to explore determinants of variation in reference intervals. Kenya is one of the three participating countries in Africa, the other ones being Nigeria and South Africa. For the Kenyan study, we recruited subjectively healthy black African adults who met the inclusion criteria as spelt out in the published protocol [13]. Participants were recruited from Nairobi which is the capital city, Kiambu county which is in central Kenya, Kisii which is in the western part of Kenya, and Nakuru county which is in the Great Rift valley. Recruitment was carried out between January and October 2015. All participants had undergone an overnight fast and written informed consent was sought from each participant after giving a written and verbal explanation of the study. Recruitment was stratified into 4 age groups: 18–29, 30–39, 40–49 and 50–65 years with almost similar numbers of males and females in each age strata. The recruitment procedure, sample handling and analysis has previously been described [14]. Briefly, all participants had measurements of blood pressure (BP), abdominal circumference and body mass index (BMI) done, samples were collected and centrifuged within 4 h after collection and stored at -80^o^c until shipment on dry ice to the Pathcare reference laboratory in South Africa. As part of the reference interval study, all participating laboratories used a common mini-panel of sera with assigned values to ensure accuracy of reported results and alignment of values if any biases were identified. Urine samples to test for haematuria and proteinuria were not collected. Serum creatinine was determined using a standardized kinetic colour test (Jaffé method) and fasting plasma glucose (FPG) by an enzymatic UV test (hexokinase method) both measured on a Beckman AU5800 (Schizuoka, Japan).

Calculation of body surface area (BSA) was done using the DuBois method [15]: BSA (m^2^) = [71.84 × weight (kg)^0.425^ × height (cm)^0.725^]/10 000..

Calculation of eGFR (mL/min/1.73 m^2^) was done using the following equations:

Re-expressed 4-v MDRD [16]

$$ 175\times {\left[\mathrm{S}\hbox{-} \mathrm{Cr}\ \left(\upmu \mathrm{mol}/\mathrm{L}\right)/88.4\right]}^{\hbox{-} 1.154}\times \mathrm{age}\ {\left(\mathrm{years}\right)}^{\hbox{-} 0.203}\times \left(0.742\ \mathrm{if}\  \mathrm{female}\right)\times \left(1.212\ \mathrm{if}\  \mathrm{African}\  \mathrm{American}\right) $$

Cockcroft-Gault normalized to 1.73m^2^ [3]

$$ \left[\left(140\times \mathrm{age}\ \mathrm{in}\  \mathrm{years}\right)\times \mathrm{weight}\ \left(\mathrm{kg}\right)\times \left(0.85\ \mathrm{if}\  \mathrm{female}\right)\times 1.73\ \left(\mathrm{m}2\right)\right]/\left[\mathrm{S}\hbox{-} \mathrm{Cr}\left(\upmu \mathrm{mol}/\mathrm{L}\right)\times 0.814\times \mathrm{BSA}\ \left({\mathrm{m}}^2\right)\right] $$

CKD-EPI for blacks [9]

$$ \mathrm{Female}\  \mathrm{with}\  \mathrm{Creatinine}\le 62\ \upmu \mathrm{mol}/\mathrm{L};166\times {\left({\mathrm{S}}_{\mathrm{cr}}/61.9\right)}^{\hbox{-} 0.329}\times {(0.993)}^{\mathrm{Age}} $$

$$ \mathrm{Female}\  \mathrm{with}\  \mathrm{Creatinine}>62\ \upmu \mathrm{mol}/\mathrm{L};166\times {\left({\mathrm{S}}_{\mathrm{cr}}/61.9\right)}^{\hbox{-} 1.209}\times {(0.993)}^{\mathrm{Age}} $$

$$ \mathrm{Male}\  \mathrm{with}\  \mathrm{Creatinine}\le 80\ \upmu \mathrm{mol}/\mathrm{L};163\times {\left({\mathrm{S}}_{\mathrm{cr}}/79.6\right)}^{\hbox{-} 0.411}\times {(0.993)}^{\mathrm{Age}} $$

$$ \mathrm{Male}\  \mathrm{with}\  \mathrm{Creatinine}>80\ \upmu \mathrm{mol}/\mathrm{L};163\times {\left({\mathrm{S}}_{\mathrm{cr}}/79.6\right)}^{\hbox{-} 1.209}\times {(0.993)}^{\mathrm{Age}} $$

CKD-EPI for other races [9]

$$ \mathrm{Female}\  \mathrm{with}\  \mathrm{Creatinine}\le 62\ \upmu \mathrm{mol}/\mathrm{L};144\times {\left({\mathrm{S}}_{\mathrm{cr}}/61.9\right)}^{\hbox{-} 0.329}\times {(0.993)}^{\mathrm{Age}} $$

$$ \mathrm{Female}\  \mathrm{with}\  \mathrm{Creatinine}>62\ \upmu \mathrm{mol}/\mathrm{L};144\times {\left({\mathrm{S}}_{\mathrm{cr}}/61.9\right)}^{\hbox{-} 1.209}\times {(0.993)}^{\mathrm{Age}} $$

$$ \mathrm{Male}\  \mathrm{with}\  \mathrm{Creatinine}\le 80\ \upmu \mathrm{mol}/\mathrm{L};141\times {\left({\mathrm{S}}_{\mathrm{cr}}/79.6\right)}^{\hbox{-} 0.411}\times {(0.993)}^{\mathrm{Age}} $$

$$ \mathrm{Male}\  \mathrm{with}\  \mathrm{Creatinine}>80\ \upmu \mathrm{mol}/\mathrm{L};141\times {\left({\mathrm{S}}_{\mathrm{cr}}/79.6\right)}^{\hbox{-} 1.209}\times {(0.993)}^{\mathrm{Age}} $$

FAS equation [11].

2–40 years; 107.3/(Scr/Q).

> 40 years; (107.3/[Scr/Q])0.988^Age ‐ 40^.

Where Scr is serum creatinine, Q is the mean or median population specific serum creatinine, Age is in years.

Classification of CKD based on eGFR was done using the 2012 Kidney Disease: Improving Global Outcomes (KDIGO) clinical practice guidelines as follows [17]:

**Table:** 

GFR category	GFR (ml/min/1.73m^2^)	Terms
G1	≥ 90	Normal or high
G2	60–89	Mildly decreased
G3a	45–59	Mildly to moderately decreased
G3b	30–44	Moderately to severely decreased
G4	15–29	Severely decreased
G5	< 15	Kidney failure

A cut off-of 75 ml/min/1.73m^2^ was also used to define reduced eGFR for participants less than 40 years of age as proposed by Pottel et al. [18]. Sample size calculation for the primary study was based on recommendations from the Clinical Laboratory Standards Institute (CLSI) which requires a minimum of 120 study participants per stratification in order to get 90% confidence limits around the upper and lower limits of a reference interval [19]. Since we had 4 age stratifications, a minimum sample size of 480 was required to achieve the primary objective for the reference interval study.

### Statistical analysis

Descriptive and inferential statistics were performed using SPSS version 20 (IBM corp, Armonk, New York, USA). Medians and interquartile ranges (IQR) were calculated for continuous variables before and after stratification based on gender. Comparison of medians or mean ranks between males and females was done using the Mann-Whitney U test. Classification of eGFR was reported as proportions. Multiple regression analysis (MRA) was also performed to determine the extent to which the CKD risk factors independently added to the eGFR regression model. Calculation of eGFR, BSA and classification of CKD was done using Microsoft excel 2010 (Redmond, USA). A *p*-value less than 0.05 was considered statistically significant.

## Results

A total of 533 participants were included comprising 260 (48.8%) males and 273 (51.2%) females. The median age, BMI and FPG between males and females wasn’t statistically different neither was eGFR except when determined using CG corrected for BSA. Males had higher serum creatinine values. Five participants didn’t have FPG assayed due to insufficient samples. A summary of the participants’ demographic and clinical descriptive statistics are shown in Table 1.

**Table 1:** Descriptive characteristics of participants

	Male (*n* = 260)		Female (*n* = 273)		Total (*n* = 533)		Male vs Female
	Median (IQR)	Min-Max	Median (IQR)	Min-Max	Median (IQR)	Min-Max	*p*-value
Age (years)	39 (18)	20–65	39 (21)	18–64	39 (20)	18–65	0.971
Height (cms)	172 (8)	156–191	160 (9)	143–191	167 (13)	143–191	0.000
Weight (kg)	74 (19)	46–116	68 (16)	38–109	70 (18)	38–116	0.000
BMI (kg/m^2^)	24.9 (5.6)	16.3–34.9	26.1 (6.3)	17.1–38.1	25.5 (5.9)	16.3–38.1	0.051
BSA (m^2^)	1.88 (0.24)	1.44–2.36	1.72 (0.20)	1.27–2.24	1.78 (0.22)	1.27–2.36	0.000
Abd. Circ. (cm)	91 (15)	65–124	86 (16)	64–115	89 (17)	64–124	0.005
BP Systolic (mmHg)	127 (18)	84–179	128 (18)	118 (20)	124 (21)	77–194	0.000
BP Diastolic (mmHg)	81 (12)	56–101	79 (14)	57–112	80 (14)	56–112	0.003
^a^FPG (mmol/L)	4.9 (0.8)	3.0–15.6	4.8 (0.7)	3.3–19.5	4.9 (0.8)	3.0–19.5	0.191
Creatinine (μmol/L)	80 (20)	50–126	61 (15)	30–93	70 (21)	30–126	0.000
4v–MDRD eGFR (mL/min/1.73m^2^)	114 (34)	61–184	118 (34)	69–251	115 (33)	61–251	0.126
4v–MDRD^b^ eGFR (mL/min/1.73m^2^)	94 (28)	50–152	97 (28)	57–207	95 (28)	50–207	0.126
CKDEPI eGFR (mL/min/1.73m^2^)	118 (25)	60–151	124 (25)	71–168	121 (25)	60–168	0.090
CKDEPI eGFR^b^ (mL/min/1.73m^2^)	102 (21)	52–130	107 (22)	62–146	105 (22)	52–146	0.061
CG eGFR^c^ (mL/min/1.73m^2^)	103 (26)	55–163	163 (45)	85–345	128 (61)	55–345	0.000
FAS eGFR (mL/min/1.73m^2^)	100 (23)	51–163	104 (25)	58–200	102 (25)	51–200	0.309

KEY: *IQR* Interquartile range, *Abd.Circ.* Abdominal circumference, *BMI* Body Mass Index, *BP* Blood pressure, *FPG* Fasting plasma glucose (^a^5 males didn’t have this test done), ^b^Not corrected for race, ^c^Corrected for body surface area

Overall, women had higher eGFR compared to men. Of the 6 equations, the 4v–MDRD equation without correction for race had the lowest median eGFR and classified the least number of participants (61.7%) as having an eGFR ≥90 mL/min/1.73m^2^ compared to 93.6% by CKD-EPI with correction for race as shown in Table 2. The 4v–MDRD equation without correction for race classified the highest percentage of participants (0.9%) as having an eGFR <60 ml/min/1.73m^2^. The number of individuals with an eGFR < 60 ml/min/1.73m^2^ was less than 1% regardless of the equation used. Using a cut off of 75 ml/min/1.73m^2^, the 4v–MDRD equation without correction for race also classified the highest proportion of participants under the age of 40 years (7.8%) as having reduced eGFR as shown in Table 2.

**Table 2:** CKD classification and correlation of eGFR with risk factors

	CG^b^	4v–MDRD	4v–MDRD^a^	CKD-EPI	CKD-EPI^a^	FAS
CKD classification based on eGFR No. (%)						
G1	478 (89.7%)	473 (88.7%)	329 (61.7%)	499 (93.6%)	433 (81.2%)	402 (75.4%)
G2	54 (10.1%)	60 (11.3%)	199 (37.3%)	34 (6.4%)	99 (18.6%)	129 (24.2%)
G3a	1 (0.2%)	0 (0.0%)	5 (0.9%)	0 (0.0%)	1 (0.2%)	2 (0.4%)
No. (%) below 40 years with eGFR< 75 ml/min/1.73m^2^ (*n* = 270)	0 (0.0%)	0 (0.0%)	21 (7.8%)	0 (0.0%)	1 (0.4%)	5 (1.9%)
eGFR (ml/min/1.73m^2^) mean (SD)						
18–29 years (*n* = 135)	149.0 (40.9)	129.5 (21.8)	106.8 (18.0)	135.3 (15.6)	117.2 (13.5)	108.9 (15.5)
30–39 years (n = 135)	136.7 (39.2)	112.1 (20.2)	92.6 (16.7)	119.1 (15.8)	103.2 (13.7)	102.9 (15.8)
40–49 years (*n* = 132)	139.8 (43.6)	117.0 (25.4)	96.6 (20.9)	117.6 (14.8)	101.8 (12.8)	106.7 (19.8)
50–65 years (*n* = 131)	120.8 (35.1)	111.9 (25.0)	92.3 (20.6)	107.6 (15.2)	93.2 (13.1)	92.2 (18.4)
Multiple correlation coefficient (R)	0.563	0.305	0.306	0.568	0.568	0.350
Unstandardized (B) coefficients (standard error)						
Age	−0.621* (0.151)	−0.322* (0.102)	−0.267* (0.084)	−0.673* (0.067)	−0.579* (0.058)	−0.403* (0.077)
BMI	7.418* (0.567)	−0.596 (0.382)	−0.491 (0.315)	−0.188 (0.252)	−0.129 (0.218)	−0.405 (0.289)
Systolic BP	−0.485* (0.140)	−0.121 (0.094)	−0.099 (0.078)	−0.116 (0.062)	−0.102 (0.054)	−0.119 (0.071)
Diastolic BP	0.237 (0.202)	0.139 (0.136)	0.115 (0.112)	0.142 (0.090)	0.124 (0.078)	0.117 (0.103)
Abd.Circ.	−2.049* (0.231)	−0.178 (0.156)	−0.148 (0.128)	−0.184 (0.103)	−0.172 (0.089)	−0.028 (0.118)
FPG	0.579 (1.170)	1.106 (0.788)	0.912 (0.650)	0.610 (0.519)	0.500 (0.450)	0.807 (0.596)

KEY: *Abd.Circ.* Abdominal circumference, *BP* Blood pressure, *BMI* Body Mass Index, *CKD* Chronic kidney disease, *FPG* Fasting plasma glucose, ^a^Not corrected for race, ^b^Corrected for body surface area, * Significant at a *p*-value <0.05

After multiple regression analysis, only age had an unstandardized coefficient (B) that was statistically significantly different from zero across all the 6 equations. Overall, the correlation between eGFR and risk factors for CKD varied depending on which equation was used. The 6 CKD risk factors evaluated had a stronger association with the CKD-EPI determined eGFRs as evidenced by higher multiple correlation coefficients as shown in Table 2.

## Discussion

The prevalence of eGFR < 60 ml/mim/1.73 m^2^ was less than 1% according to the 6 equations evaluated in this study which is reflective of the deliberate recruitment of healthy individuals for the reference interval study. In a randomly selected rural and urban adult population in Cameroon, Kaze et al. found that 10.9% had a CKD-EPI determined eGFR < 60 ml/min/1.73m^2^ with this being more common in the urban population [20]. Both the CKD-EPI and 4v–MDRD equations with correction for race did not classify any participant as having a reduced eGFR in our study largely comprised of an urban population. Assuming that a healthy population should predominantly have an eGFR > 90 ml/min/1.73m^2^, the CKD-EPI equation with correction for race classified more participants into stage G1 suggesting that it could be a more ideal equation when estimating eGFR in an asymptomatic population not known to have risk factors for CKD. Unlike the MDRD equation which was derived from individuals with CKD, CKD-EPI was derived from a heterogeneous population that included healthy individuals [9]. Rule et al. used different equations to screen for CKD in a general population and found that equations derived from a CKD population gave higher estimates of CKD prevalence compared to equations derived from a population that included healthy individuals [21]. It is therefore not surprising that the CKD-EPI equation classified less participants as having a reduced eGFR compared to the 4v–MDRD equation.

There has been a debate as to the suitability of the 60 ml/min/1.73m^2^ eGFR cut off for defining CKD in young adults and the elderly. Pottel et al. suggested that a cut off of 75 ml/min/1.73m^2^ would be more ideal in young adults under 40 years of age given that one would require a serum creatinine almost 1.8 times above the mean value for this population to achieve an eGFR less than 60 ml/min/1.73m^2^ making this cut off quite insensitive to early increments in serum creatinine [18]. Gharbi et al. demonstrated that in an Arabic-Berber adult population in Morocco, using a cut off of 60 ml/min/1.73m^2^ resulted in under diagnosis of CKD in younger adults and over diagnosis in the elderly population [22]. In our study, based on a cut off of 75 ml/min/1.73m^2^, no one under the age of 40 years had reduced eGFR when determined using CG corrected for BSA, 4v–MDRD or CKD-EPI equations corrected for race. The FAS equation that can be applied across a wide age spectrum classified 5 (1.9%) individuals less than 40 years of age as having a reduced eGFR compared to 21 (7.8%) for the 4v–MDRD equation without correction for race. In the absence of mGFR, testing for albuminuria or demonstrating persistence of reduced eGFR for at least 3 months, it is difficult to conclude which individuals truly had CKD and which equation was correctly classifying them as such.

We determined eGFR correlation with known risk factors for CKD such as age, BMI, BP, glycaemia and abdominal circumference. We assumed that in the absence of mGFR, the multiple correlation coefficient would serve as indirect proof of appropriateness for routine reporting of eGFR in an asymptomatic population as it is a measure of linear association between the predicted eGFR after factoring in the CKD risk factors and eGFR determined using the various equations. The CKD-EPI and CG corrected for BSA equations had the highest multiple correlation coefficients suggesting that the the model best fitted these equations. However, given that age is one of the components of the CG equation and both height and weight are used in the calculation of BMI and BSA, there is a possibility that the CG multiple correlation coefficient after correction for BSA is inaccurate due to possible collinearity. Age remained the only risk factor that showed a statistically significant linear association with eGFR across all eGFR equations. Matsha et al. demonstrated that age, gender and known history of hypertension were determinants of CKD stage 3–5 in a mixed ancestry population in South Africa. Surprisingly, this was not the case for FPG, BMI, systolic and diastolic BP. However, this study did not correlate eGFR with these potential risk factors but rather used logistic regression to determine the odds of having a reduced eGFR [23]. Kaze et al. concluded that in a rural and urban population in Cameroon, elevated systolic BP, presence of hypertension and diabetes were the main predictors of albuminuria and CKD [20]. In the Democratic Republic of Congo, a study by Sumaili et al. found that age above 65 years and hypertension were independently associated with increased risk of CKD stage 3–5 [24].

The issue of whether or not one should correct for the black African race when using eGFR equations has not been settled. Deventer et al. concluded that correction for the African race was not necessary when using the 4v–MDRD equation after they compared eGFR with mGFR in 100 black African patients with CKD and found a greater bias after correcting for race [5]. The mean weight of the participants in this study was 69.5 kg with a BSA of 1.76 m^2^ compared to a weight of 79.6 kg and BSA of 1.91 m^2^ for the MDRD population which included only 197 (12.1%) African Americans [25]. The adjustment for the African race is extrapolated from the African-American MDRD study population who had a higher mean weight and BSA compared to the Caucasian population necessitating a correction of the original 4v–MDRD eGFR equation by a factor of 1.18 that was further adjusted to 1.212 after standardization of the creatinine assay. Since serum creatinine levels are affected by muscle mass, it is not surprising that the 4v–MDRD equation without correction for race better approximated mGFR in the South African population given a closer similarity in weight and by extension muscle mass of their study population to the Caucasian population used in deriving the equation. A study carried out in Ghana in a rural population of black Africans also concluded that correction for race was unnecessary for both the 4v–MDRD and CKD-EPI equations. This study compared eGFR to CrCl and the mean weight of the participants was 54.4 kg [6]. Flamant et al. compared CKD-EPI and 4v–MDRD derived eGFR to mGFR in African Europeans originating from West Africa and concluded that a correction factor of 1.08 was required. However, most of the patients in this study had CKD with a mean mGFR of 57.6 ml/min/1.73m^2^ [26]. Delanaye et al. has argued that the correction factor for race when using the 4v–MDRD or CKD-EPI equations should vary depending on whether it is being applied in individuals with CKD and an eGFR < 60 ml/min/1.73m^2^ or healthy subjects due to the inherent difference in how these 2 populations handle serum creatinine even within the same race [27]. In our study we didn’t compare eGFR to either mGFR or CrCl but rather compared the proportion of healthy black Africans classified as having a reduced or normal eGFR across the different equations and eGFR correlation with a prediction model comprising risk factors for CKD. Since the correction factor for race for both 4v–MDRD and CKD-EPI is greater than one, it is not surprising that correction for race resulted in higher eGFRs. However, the CKD EPI equation had a higher median eGFR and proportion of individuals with eGFR > 90 ml/min/1.73m^2^ compared to 4v–MDRD or FAS as well as better correlation with risk factors for CKD. This is not surprising as CKD-EPI is more accurate than the 4v–MDRD equation in individuals with a GFR >60 ml/min/1.73m^2^ and would be expected to be more appropriate for the subjectively healthy population recruited in this study [9]. The presence of a larger proportion of healthy individuals in the CKD-EPI dataset makes it more suitable for reporting eGFR in healthy subjects [21]. The FAS equation was developed to overcome the potential differences in eGFR associated with adoption of different equations. The equation incorporates population normalized serum creatinine levels and therefore gets rid of the need for further age, gender and race correction. This equation has been shown to be less biased than the CKD-EPI equation when applied on data sets largely comprised of a Caucasian population and therefore requires validation in an ethnic black African population [11]. In our study, the FAS determined eGFR had similar correlation coefficients with CKD risk factors as the 4v–MDRD equation and demonstrated a similar pattern in change of mean eGFR with age where the mean eGFR in the age group 40–49 years was higher than in the age group 30–39 years. Of the 6 equations, only the CKD-EPI derived mean eGFRs showed a consistent decline with increase in age across the 4 age stratifications in line with the expected reduction of eGFR associated with progressive loss of nephrons above the age of 30 years [28].

The median eGFR for female participants calculated using the various equations was consistently higher than male participants in our study though this difference was not statistically significant except for eGFR determined using CG corrected for BSA. This is in contrast to the Ghanaian study by Eastwood et al. where the female participants had a lower mean eGFR when determined using both the 4v–MDRD and CKD-EPI equations with and without correcting for race but not when CG corrected for BSA was used. Similar to our study, the Ghanaian women had a higher BMI but lower weight and blood pressure compared to the men [6]. No obvious explanation is forthcoming for the relatively better eGFR seen in women compared to men in our study. The Ghanaian rural population had a higher percentage (13.2%) of individuals with reduced eGFR (< 60 ml/min/1.73m^2^) compared to < 1% in our study population which can partially be explained by the fact that we recruited a younger population ranging from 18 to 65 years compared to 40–75 years for the Ghanaian population with 50.8% of our study population being under the age of 40 years. We also had a strict inclusion and exclusion criteria to ensure that as far as possible only healthy individuals were recruited.

A major limitation of our study is that we didn’t measure GFR and as such our comparison of various eGFR equations is not validated against a gold standard. In the absence of mGFR, we chose to use correlation with a prediction model of markers known to increase risk of CKD but are not included in the eGFR equations as a basis of comparison. Our assumption was that equations whose eGFR calculations best correlated with these risk factors were potentially better equations to be used when screening for CKD in an asymptomatic black African population. This is not equivalent to mGFR and therefore limits the extent to which conclusions can be made as to which equation is better. Another major limitation is that we didn’t carry out any test for albuminuria neither did we repeat serum creatinine measurements after 3 months hence we cannot accurately comment on the prevalence of CKD in this population. Gharbi et al. found that 32% of the subjects classified as having CKD stage 3a and 7.4% of those classified as 3b had an eGFR > 60 ml/min/1.73 m^2^ when reinvestigated after 3 months or longer [22]. However, the proportion of participants with an eGFR <60 ml/min/1.73 m^2^ in our study was less than 1% hence the risk of over diagnosing CKD in this healthy population was extremely low. What is more likely is that we may have under diagnosed CKD due to failure to test for albuminuria. We also assumed that diabetes and hypertension are the commonest risk factors for CKD in our population which may not necessarily be the case as demonstrated by Stanifer et al. in Northern Tanzania where 49.1% of individuals with CKD didn’t have hypertension, diabetes or HIV [29]. We also didn’t measure cystatin C which has been shown by Meeusen et al. to significantly improve eGFR estimation across different patient groups including healthy kidney donors when incorporated into the CKD-EPI equation [30].

## Conclusion

The CKD-EPI equation with correction for race classified the highest proportion of asymptomatic healthy black Africans as having an eGFR >90 ml/min/1.73m^2^ and its eGFR better correlated with risk factors for CKD. For this reason, we recommend the use of the CKD-EPI equation with correction for race for routine reporting of eGFR in an asymptomatic black African population with a low prevalence of risk factors for CKD. Comparing eGFR to mGFR would be the best way to determine the most appropriate equation for CKD screening in an asymptomatic black African population. However, measurement of GFR is a complex and expensive process which serves as a barrier for the validation of eGFR equations especially in sub Saharan Africa. In the absence of mGFR, other ways of evaluating the potential utility and performance of eGFR equations in routine screening for CKD in asymptomatic populations need to be evaluated.

## Abbreviations

4v–MDRD: Re-expressed 4 variable modified diet in renal disease
Abd.Circ.: Abdominal circumference
BMI: Body mass index
BP: Blood pressure
BSA: Body surface area
CG: Cockcroft-Gault
CKD: Chronic kidney disease
CKD-EPI: Chronic kidney disease epidemiology collaboration
CrCl: Creatinine clearance
eGFR: Estimated glomerular filtration rate
FAS: Full age spectrum
FPG: Fasting plasma glucose
GFR: Glomerular filtration rate
IQR: Interquartile range
KDIGO: Kidney Disease: Improving Global Outcomes
Max.: Maximum
MDRD: Modified diet in renal disease
mGFR: Measured glomerular filtration rate
Min.: Minimum
SD: Standard deviation
